# *N*-Acetylglucosamine Inhibits LuxR, LasR and CviR Based Quorum Sensing Regulated Gene Expression Levels

**DOI:** 10.3389/fmicb.2016.01313

**Published:** 2016-08-23

**Authors:** Önder Kimyon, Zehra İ. Ulutürk, Shashidhar Nizalapur, Matthew Lee, Samuel K. Kutty, Sabrina Beckmann, Naresh Kumar, Mike Manefield

**Affiliations:** ^1^School of Biotechnology and Biomolecular Sciences, University of New South Wales, SydneyNSW, Australia; ^2^School of Chemistry, University of New South Wales, SydneyNSW, Australia

**Keywords:** chitin, quorum sensing, acetyl glucosamine, *N*-acylhomoserine lactone, chitinase

## Abstract

*N*-acetyl glucosamine, the monomer of chitin, is an abundant source of carbon and nitrogen in nature as it is the main component and breakdown product of many structural polymers. Some bacteria use *N*-acyl-L-homoserine lactone (AHL) mediated quorum sensing (QS) to regulate chitinase production in order to catalyze the cleavage of chitin polymers into water soluble *N*-acetyl-D-glucosamine (NAG) monomers. In this study, the impact of NAG on QS activities of LuxR, LasR, and CviR regulated gene expression was investigated by examining the effect of NAG on QS regulated green fluorescent protein (GFP), violacein and extracellular chitinase expression. It was discovered that NAG inhibits AHL dependent gene transcription in AHL reporter strains within the range of 50–80% reduction at low millimolar concentrations (0.25–5 mM). Evidence is presented supporting a role for both competitive inhibition at the AHL binding site of LuxR type transcriptional regulators and catabolite repression. Further, this study shows that NAG down-regulates CviR induced violacein production while simultaneously up-regulating CviR dependent extracellular enzymes, suggesting that an unknown NAG dependent regulatory component influences phenotype expression. The quorum sensing inhibiting activity of NAG also adds to the list of compounds with known quorum sensing inhibiting activities.

## Introduction

Chitin, a polymer of linked amino sugar subunits (β-1,4-linked *N*-acetyl-D-glucosamine; NAG), is the second most abundant biological polymer on Earth (most abundant in the marine environment) with global production estimated at 10^11^ tons per year (Gooday, 1990; Souza et al., 2011). The fact that chitin does not accumulate in the environment indicates that chitin biodegradation (turnover) rates are as immense as production rates and that chitin biodegradation plays a major role in nutrient cycling (linking carbon and nitrogen cycles) and hence maintenance of life globally (Gooday, 1990). Extracellular chitinase enzymes catalyze the cleavage of the chitin polymer into water soluble oligomers and thereafter into dimers [(NAG)_2_] and monomers (NAG) that are taken up by microorganisms and catabolised intracellularly (Gooday, 1990; Keyhani and Roseman, 1999). Regulation of chitinase gene expression is complex and varied between even closely related bacterial strains, but chitinase genes are generally upregulated upon detection of chitin or chitin degradation products and downregulated by catabolite repression (Ulhoa and Peberdy, 1991; Techkarnjanaruk et al., 1997; Keyhani and Roseman, 1999). Additionally, chitinase production is controlled in some instances by *N*-acyl-L-homoserine lactone (AHL) mediated quorum sensing (Chernin et al., 1998; Hentzer et al., 2002; Christensen et al., 2003; Defoirdt et al., 2010; Chong et al., 2012).

It is common for many bacteria to regularly encounter NAG, the monomer of the chitin polymer, in various environments given the abundance of NAG sources in nature (Park and Uehara, 2008). Therefore, bacterial and eukaryotic cells developed mechanisms by which NAG regulates virulence properties of microbes and host cells. For example, NAG inhibits the production of type-1-fimbriae in *Escherichia coli*, thereby promoting infection (Sohanpal et al., 2004). NAG also modulates production of antimicrobials and toxins in *Pseudomonas aeruginosa* PA14 via a peptidoglycan sensing protein (Korgaonkar et al., 2013). Additionally, NAG induces chitinase activity in *Pseudoalteromonas* and *Vibrio* species while it reduces chitinase activity in *Serratia marcescens* and *Trichoderma harzianum* in a strain dependent manner (Monreal and Reese, 1969; Ulhoa and Peberdy, 1991; Chernin et al., 1998; Bhowmick et al., 2007).

*N*-Acyl-L-Homoserine Lactones mediated quorum sensing (QS) is a gene expression mechanism involving a transcriptional link between the extracellular accumulation of AHL and genes encoding phenotypes such as biofilm formation, bioluminescence, virulence factor production and exoenzyme production (Bassler, 1999; Williams et al., 2007). AHL production (AHL synthase, LuxI homologue) and response (AHL response regulator, LuxR homologue) is widespread amongst the α, β, and γ Proteobacteria (Manefield and Turner, 2002), which are generally highly abundant in nature. Recent studies have shown that AHL-mediated QS activity is not restricted to Proteobacteria but also observed in archaea and marine bacteria including *Flavobacterium* spp. and *Tenacibaculum maritimum* which belong to Bacteroidetes (Romero et al., 2010; Zhang et al., 2012; Twigg et al., 2014). AHL producing bacteria have been isolated from a wide variety of environments including soil, plants, animals, fresh water, marine water, and activated sludge in wastewater treatment plants.

QS mechanisms are attractive targets to interfere with bacterial virulence. It is evident that disruption of QS activities of pathogenic bacteria significantly reduces their virulence activities (LaSarre and Federle, 2013). Therefore, many studies have focussed on designing synthetic QS agonists and antagonists for inhibition of QS activities dependent on LuxR type regulators (Welsh et al., 2015; Nizalapur et al., 2016). Moreover, several types of QS inhibitor compounds are known to be produced by eukaryotes and prokaryotes. These compounds target the QS systems by different mechanisms including enzymatic degradation of AHLs, interference with the stability or function of AHL receptor and/or synthase proteins and inhibition of the production of AHLs (Givskov et al., 1996; Zhang et al., 2002; Gao et al., 2003; Kalia, 2013). In addition to known QS inhibitors, recent studies point out the importance of nutritional conditions on regulation of QS system within Gram-negative bacteria. It has been reported that specific carbon sources mediate QS activity and swarming motility in *P. aeruginosa* biofilm formation (Shrout et al., 2006). Similarly, glucose treated *Aeromonas hydrophila* cultures downregulate QS regulated phenomena such as biofilm formation and virulence (Jahid et al., 2013). On the other hand, starvation regulates QS signaling and represses cell growth in *P. aeruginosa* and *Bacillus subtilis* which triggers entry into the stationary phase (You et al., 1998; Lazazzera et al., 1999). The cylic AMP (cAMP) cAMP receptor protein (CRP) regulates numerous genes as both an activator and repressor (Kolb et al., 1993). Its role in regulating carbon metabolism through carbon catabolite repression of different bacteria such as *E. coli* and *B. subtilis* has been well characterized (Gorke and Stulke, 2008). It is also reported that CRP is required to control transcription of *luxI*/*luxR* homolog genes which encode QS signal receptor (Albus et al., 1997; Dunlap, 1999; Liang et al., 2007).

The objective of this study was to investigate the relationship between the chitin monomer (NAG) and quorum sensing activities of AHL reporter strains, and for this purpose, cultures were treated with various concentrations of NAG and the effect of NAG was compared to the related molecules glucose and glucosamine (GlcN). The role of catabolite repression was also examined.

## Materials and Methods

### Bacterial Strains and Culture Conditions

The bacterial strains used in this study are shown in Supplementary Table S1. Media supplements to AB minimal media (Clark and Maaløe, 1967) used for culturing were as follow; 0.2% tryptone, 0.4% yeast extract for *Chromobacterium violaceum* CV026, 0.2% tryptone, 0.1% lactate for *E. coli* MT102, and 0.2% tryptone, 0.1% yeast extract for *P. aeruginosa* MH602. Strains were grown at either 30°C (strain CV026) or 37°C (strains MT102 and MH602) with shaking at 150 rpm. *N*-acyl-L-homoserine lactones including *N*-hexanoyl-L-homoserine lactone (HHL), *N*-(3-oxo-hexanoyl)-L-homoserine lactone (OHHL), *N*-(3-oxo-dodecanoyl)-L-homoserine lactone (OdDHL; Sigma-Aldrich) were dissolved in MeOH and stored as 20 mM stocks at -20°C. Growth experiments commenced at an OD_600_ of 0.01 with reading taken at 30-min intervals using a multiwell plate reader (Ensight Plate Reader, PerkinElmer).

### Screening of AHL-Mediated GFP(ASV) Production

AHL dependent GFP expressing *E. coli* MT102 (pJBA132) and *P. aeruginosa* MH602 strains were cultured overnight with appropriate AB media described above. Strains were diluted (1:15) and 100-μl aliquots were dispensed to flat bottom 96-well plate wells (Sarstedt Australia). *E. coli* MT102 cultures were supplemented with 10 nM OHHL and air dried prior to addition of cultures. No OHHL was added into negative control culture. Cultures were also supplemented with NAG, GlcN or glucose within the range of 0.25–2 mM. Positive control culture was supplemented with no extra carbon sources. Plates were sealed with self-adhesive microplate sealers (TopSeal-A, PerkinElmer) to allow air diffusion and to prevent condensation. Cultures were incubated at 37°C with shaking at 150 rpm overnight. Fluorescence (excitation, 485 nm; emission, 535 nm) and OD_600_ of cultures were measured by a multiwell plate reader (Ensight Plate Reader, PerkinElmer). The ratio of the fluorescence to OD_600_ (Fluorescence/OD_600_) represented the unit fluorescence activity. All cultures were prepared in triplicates and experiments were repeated at least three times.

### Violacein Extraction and Quantification

Violacein production by *C. violaceum* CV026 was quantified as previously described (Blosser and Gray, 2000). Briefly, cultures were supplemented with 100 nM HHL and treated with NAG or GlcN within the range of 0.5–5 mM. All cultures were incubated at 30°C with shaking at 150 rpm over 20 h. Supernatants from 800 μL aliquots were discarded and cells were lysed by adding 200 μl of 10% sodium dodecyl sulfate and incubated at 25°C for 5 min. Violacein was extracted by adding 1 ml of water-saturated butanol and absorbance was taken at 585 nm. Negative control culture was treated with no HHL and carbon source. Positive control culture was supplemented with no extra carbon source. The OD_660_ of each culture was measured prior to violacein extraction. Absorbance readings were taken by a multiwell plate reader (Ensight Plate Reader, PerkinElmer). The ratio of the violacein absorbance (585 nm) to OD_660_ represented the unit violacein activity. All cultures were prepared in triplicates and experiments were repeated at least three times.

### Growth Experiments

*C. violaceum* CV026, *E. coli* MT102 and *P. aeruginosa* MH602 strains were cultured in AB medium supplemented with 0.01% yeast extract and 20 mM of NAG, GlcN or glucose. Control cultures lacked an additional carbon source. Initial OD_600_ of all cultures was adjusted to 0,01 and optical density readings for MT102 and MH602 were taken at 30-min intervals whereas the OD_600_ of CV026 was measured after 20 h incubation by using a multiwell plate reader (Ensight Plate Reader, PerkinElmer).

### RNA Transcript Analysis by qRT-PCR

Transcriptional differences of QS regulated genes were analyzed by qRT-PCR as follows. Total RNA was extracted from cultures at mid-logarithmic growth phase using RNeasy Mini Kit (QIAGEN). Synthesis of cDNA with primers listed in Supplementary Table S1 was performed with AVM Reverse Transcriptase (Promega) according to manufacturer’s instructions. Resulting cDNA was used as template in subsequent qPCR amplification of *gfp(ASV)* and violacein biosynthetic genes including *vioA, vioC, vioD*. Specific transcripts were verified with primers (Supplementary Table S1) designed using the NCBI primer-designing tool. Transcript abundance was log_5_ transformed and normalized to the 16S rRNA transcript abundance.

### Ligand-Receptor Docking Studies

Possible binding sites and poses of the compounds within quorum sensing receptors LasR, TraR, and CviR (Zhang et al., 2002; Bottomley et al., 2007; Chen et al., 2011) were predicted by docking compounds into receptors LasR (PDB code, 2UV0), TraR (PDB code, 1L3L) and CviR (PDB codes, 3QP4, 3QP5) using Ligand Docking (GOLD; Cambridge Crystallography Data Centre, UK) in its implementation through the Discovery Studio (Accelrys) interface. Hydrogen atoms were added to all ligands and the receptor prior to performing the docking runs. All ligands were also minimized under the CHARMm forcefield. The binding pocket was defined from the binding site of the agonist or antagonist in the crystal structure. The number of docking runs was set to 10, the “Detect Cavity” and “Early Termination” options were set to be “False.” All other parameters were left at their default values. Gold scores, hydrogen bonds, and π-interactions of the ligands were analyzed for the first pose with the highest Gold score.

### Chitinase Activity Assays

Extracellular chitinase activity was monitored through a commercially available chitinase assay kit (CS0980, Sigma-Aldrich). Cultures were grown overnight in AB medium supplemented with 0.2% tryptone, 0.1% yeast extract. Chitin flakes (1%) were supplemented to growth medium when it is required. Cell-free supernatants of cultures were incubated with 4-Nitrophenyl-*N*-acetyl-β-D-glucosaminide (1 mg/ml) and chitinase units were calculated according to manufacturer’s instructions. Results represent the chitinase units/per cell. Absorbance (405 nm) was measured by a multiwell plate reader (Ensight Plate Reader, PerkinElmer). All cultures were prepared in triplicates.

### Statistical Analysis

GraphPad Prism 5 software was used to apply Student’s *t*-test where necessary to determine the significant differences observed in the assays. *P*-value lower than 0.01 (*P* < 0.01) indicated significant differences.

## Results

### The Chitin Monomer *N*-Acetylglucosamine Inhibits QS Activity

**FIGURE 1:**
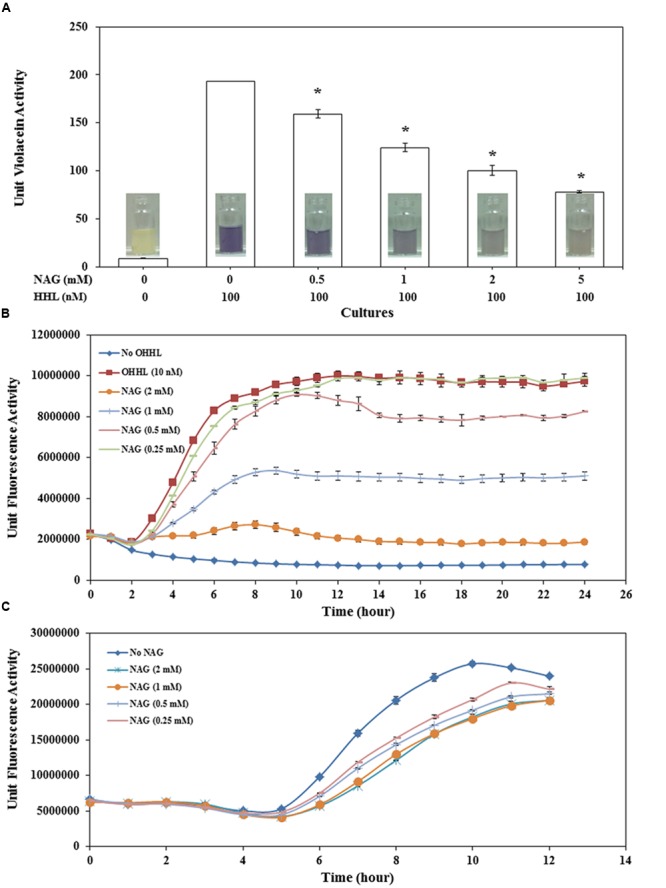
**Concentration dependent effect of NAG on **(A)** CviR dependent violacein production by *C. violaceum* CV026 with violacein extracted after 20 h incubation, **(B)** LuxR dependent specific fluorescence activity of *E. coli* MT102 (*p*JBA132) over time in the presence of 10 nM OHHL and **(C)** LasR dependent specific fluorescence activity of *P. aeruginosa* MH602.** All cultures were in triplicate. Error bars represent standard deviation. Asterisks indicate the significant differences in comparison to control samples (*P* < 0.01).

The impact of NAG on AHL-mediated QS was analyzed with CviR-based *C. violaceum* CV026, LasR-based *P. aeruginosa* MH602 and LuxR-based *E. coli* MT102 reporter strains. AHL reporter strains were treated with NAG ranging from 0.25 mM to 5 mM. AHL deficient strains were supplemented with AHLs required for optimum QS activity (100 nM HHL and 10 nM OHHL for CV026 and MT102, respectively). AHL dependent reporter activity of all three strains was significantly (*P* < 0.01) reduced in the presence of NAG (**Figure 1**).

Violacein production by CV026 decreased in a concentration-dependent manner with 0.5 mM NAG reducing production by ∼18% and 5 mM reducing production by ∼60% (**Figure 1A**). Similar results were observed with the *C. violaceum* wild type strain (data not shown). QS activity of *E. coli* MT102 exhibited higher sensitivity to NAG in comparison to CV026 (**Figure 1B**). The minimum and maximum inhibitory concentrations of NAG on LuxR-based QS activity were 0.5 mM (∼15.2% inhibiton) and 2 mM (∼81% inhibition), respectively. No recovery was observed in QS activities of both *E. coli* MT102 and *C. violaceum* CV026 over time. In *P. aeruginosa* MH602, maximum inhibition (47%) was observed at mid-log growth phase (after 7 h incubation) in 2 mM NAG supplemented cultures. The minimum inhibitory concentrations were in the 0.25–0.5 mM range (**Figure 1C**). In contrast to strains MT102 and CV026, inhibition of QS activity in strain MH602 reversed at the early stationary growth phase. Isothermal titration calorimetry (ITC) was used to test for a direct interaction between AHLs and NAG. No interactions were observed (data not shown). Millimolar concentrations of NAG are commonly observed in nature suggesting that in such environments AHL-mediated gene expression will be repressed (Gorke and Stulke, 2008; Eisenreich et al., 2010; Frimmersdorf et al., 2010; Jagmann et al., 2012).

**FIGURE 2:**
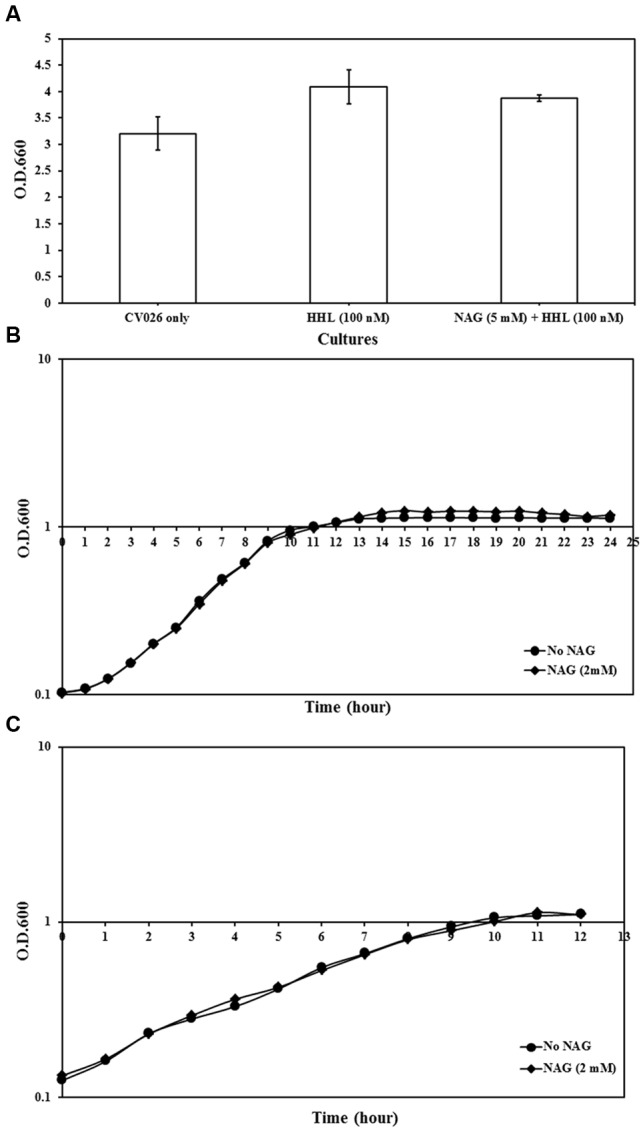
**Growth analysis of reporter strains cultured in AB minimal medium supplemented with media supplements and appropriate concentrations of NAG. (A)** OD_600_ of *C. violaceum* CV026 measured after 20 h incubation, **(B)** growth curve of *E. coli* MT102 measured at 1-h intervals during 24 h and **(C)** growth curve of *P. aeruginosa* MH602 measured at 1-h intervals during 12 h. Control cultures were supplemented with no NAG. All cultures were in triplicates. Error bars represent standard deviation.

The impact of NAG on growth rates of analyzed strains was examined. All three strains grew to cell concentrations similar to untreated cultures (**Figure 2**). This indicates that NAG does not inhibit AHL mediated gene expression through a general toxicity or global inhibition of gene expression.

### *N*-Acetylglucosamine Downregulates Transcription of QS Regulated Genes

**FIGURE 3:**
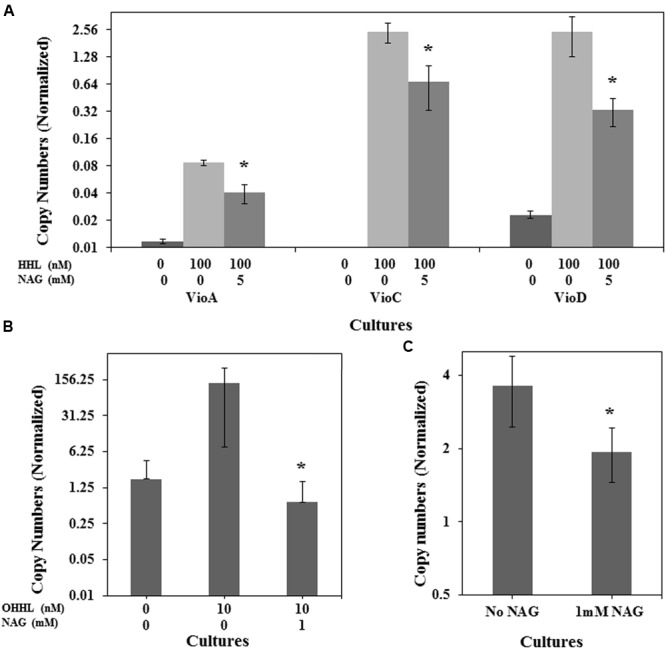
**NAG down regulates QS regulated transcriptional activity.** Transcriptional levels of **(A)** the *C. violaceum* CV026 CviR-HHL regulated *vioA, vioC*, and *vioD* genes in the presence of 100 nM HHL and 5 mM NAG, **(B)** the *E. coli* MT102 LuxR-OHHL regulated *gfp*(ASV) gene in the presence of 10 nM OHHL and 1 mM NAG and **(C)** the *P. aeruginosa* MH602 LasR-OdDHL regulated *gfp(ASV)* gene in the presence of 1 mM NAG. Cultures were sampled at mid-log growth phase for mRNA extractions. All cultures were in triplicate. Error bars represent standard deviation. Asterisks indicate the significant differences in comparison to control samples (*P* < 0.01).

To determine if NAG was exerting its effect on the AHL bioassays at a transcriptional level, three mRNA transcripts encoding violacein production in *C. violaceum* CV026 (*vioA, vioC*, and *vioD*) and the mRNA transcript encoding the green fluorescent protein in *E. coli* (pJBA132) and *P. aeruginosa* MH602 were quantified in the presence and absence of NAG. RNA was extracted from cultures in mid-log growth phase and specific transcripts quantified by RT-qPCR. Transcription levels of *vioA, vioC*, and *vioD* were reduced twofold, fourfold, and eightfold, respectively, by 5 mM NAG (**Figure 3A**) corresponding reasonably well to the threefold reduction in violacein production at this concentration (**Figure 1A**). Similarly, transcription of *gfp(ASV)* in *P. aeruginosa* MH602 was reduced twofold by 1 mM NAG (**Figure 3C**) corresponding well to the 1.6-fold decrease in fluorescence observed after an 8 h incubation at this concentration (**Figure 1C**). With *E. coli* MT102 (pJBA132) the twofold drop in fluorescence was associated with a 200-fold drop in transcript abundance in the presence of 1 mM NAG (**Figure 3B**). The reason for this is unclear. It is possible that GFP activity is compensated for at a post-transcriptional level. Overall, these data suggest that NAG down-regulates transcription from AHL dependent promoters.

### *N*-Acetylglucosamine Imposed Inhibition Compared with Glucose and Glucosamine

**FIGURE 4:**
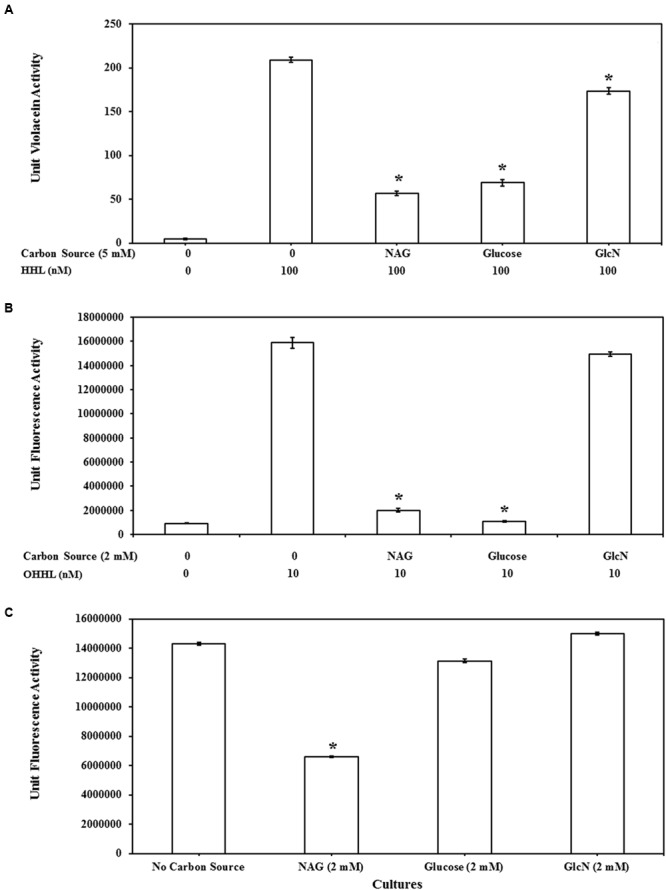
**Comparison of QS inhibitory effects of NAG with glucosamine (GlcN) and glucose at 5 mM on **(A)** CviR dependent violacein production with exogenous addition of 100 nM HHL and violacein extraction after 20 h batch incubation, at 2 mM **(B)** LuxR dependent GFP production with exogenous addition of 10 nM OHHL and measurement of fluorescence at 20 h and **(C)** LasR dependent GFP production without exogenous AHL addition, fluorescence measurement 8 h.** All cultures were in triplicate. Error bars represent standard deviation. Asterisks indicate the significant differences in comparison to control samples (*P* < 0.01).

Transcription of many QS regulated genes is also controlled by catabolite repression via cAMP and the CRP (Dunlap and Greenberg, 1988; Albus et al., 1997; Liang et al., 2007). To examine the susceptibility of the QS assays to catabolite repression the impact of related molecules GlcN and glucose on AHL mediated gene expression was also examined. The impact of glucose, GlcN and NAG on QS activity of CV026, MT102, and MH602 is represented in **Figures 4A–C**, respectively. Whilst GlcN and glucose displayed inhibitory activity the effects were reporter strain specific and less pronounced (**Figure 4**). For example the effect of glucose was similar to NAG in the LuxR dependent assay (**Figure 4B**) while no inhibition was observed in the LasR dependent assay (**Figure 4C**) suggesting catabolite repression plays a role in NAG inhibition of LuxR dependent transcription but not in LasR dependent transcription. This result is correlated with the fact that glucose is a non-preferred carbon source in *P. aeruginosa* and uptake of glucose has no effect on cAMP levels in *P. aeruginosa* (Rojo, 2010). Overall the inhibitory effects of GlcN were weak compared to NAG, with no effect observed in the LasR dependent (**Figure 4C**) assay and limited effects (6–17% reduction) observed in the LuxR and CviR dependent assays at the highest concentration tested (2 mM; **Figures 4A,B**). These data suggest that NAG exerts an effect on the activity of AHL dependent promoters through catabolite repression but that catabolite repression does not explain the inhibitory effect of NAG completely.

### Modeling Interactions with AHL Binding Sites

Bacterial cells import NAG through phosphotransferase systems that phosphorylate NAG as it enters the cell (Plumbridge, 1990). Whilst there are only vague structural similarities between AHLs and NAG (the amide bond and disposition of the ring oxygen) we considered the possibility of NAG and phosphorylated derivatives of NAG (NAG-1-phosphate and NAG-6-phosphate) interfering with AHL docking to LuxR type regulatory proteins. This might explain the inhibitory activities. With respect to size NAG has a molecular weight (mwt) of 221.21, phosphorylated NAG has a mwt of 301.19 and AHLs have mwts in the range of 213.23–297.39 (OHHL to OdDHL, respectively). With respect to polarity, the predicted log *P*-values (negative values indicate higher polarity) for NAG, NAG-1-phosphate and NAG-6-phosphateare -2.375, -2.850 and -3.699, respectively, whilst the log *P*-value for AHLs range from 0.19 for OHHL to 3.15 for OdDHL indicating NAG ligands are more water soluble or more polar than AHLs are.

**FIGURE 5:**
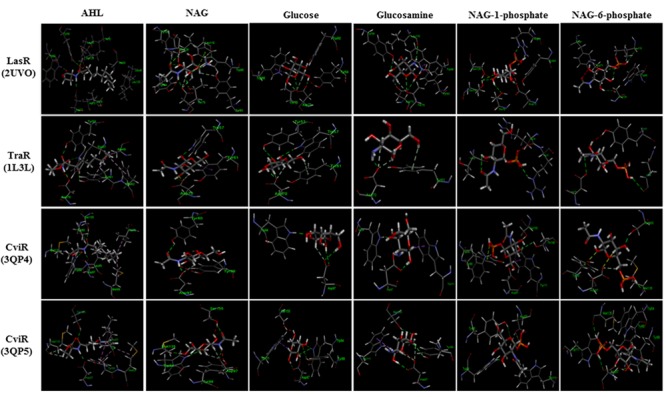
**Modeled docking poses for AHL binding sites of LasR, TraR and CviR (the latter in agonist and antagonist binding conformations) and NAG, glucosamine, glucose, NAG-1-phosphate, NAG-6-phosphate or the cognate AHLs as ligands.** All ligands could occupy the AHL binding pockets and showed hydrophilic interactions in general. Hydrogen bond interaction is shown as green dashed lines. NAG formed 40–80% of the hydrogen bonds formed by cognate AHLs and additional hydrogen bonds unique to NAG in the case of LasR.

Docking studies were performed using the AHL binding sites of LasR, TraR, and CviR (the latter in agonist and antagonist binding conformations) and cognate AHLs or NAG, phosphorylated NAG, glucose or glucosamine as ligands (**Figure 5**). **Table 1** reports the Gold Score Fitness of the ligand-receptor docking relating to binding affinity and the interactions incumbent in the selected pose. Whilst the fitness scores of NAG, glucose, glucosamine and phosphorylated NAGs are lower than the cognate AHLs for each receptor protein surprisingly all compounds have affinity for AHL binding sites. For all three receptor proteins, the fitness score for phosphorylated NAG ligands are higher than for NAG, glucose and glucosamine. For reference, there are non-cognate AHLs that have lower Gold Fitness Scores than NAG for each of the AHL receptor proteins modeled (data not shown).

**Table 1:** Gold score fitness values and interactions for NAG and other ligands docking with LasR, TraR and CviR.

QS Receptor (PDB code)	Compounds	Gold Score Fitness	Interaction from selected pose		
			H Bond	Π and Hydrophobic	Unfavorable
**LasR (2UV0)**	OdDHL	61.7	Asp73, Tyr56, Arg61, Ser129, Tyr93, Trp60	Trp88	Tyr64
	*N*-acetyl glucosamine-6-phospate	52.1	Tyr64, Ser129, Asp73, Leu110	Asp73, Trp88	_
	*N*-acetyl glucosamine-1-phospate	52.4	Arg 61, Thr115, Thr75, Asp73, Tyr64	Tyr64	_
	*N*-acetyl glucosamine	42.3	Asp 73, Tyr56, Arg61, Ser129, Tyr93, Thr75, Trp88, Leu110	Trp88	_
	Glucose	41.9	Asp 73, Tyr56	Trp88	_
	Glucosamine	40.1	Asp 73, Tyr56, Tyr93, Thr75, Trp88, Leu110	Trp88	_
**TraR (1L3L)**	OHL	61.1	Trp57, Asp70, Tyr53, Tyr61	Ala40, Tyr61, Phe62	Asp70
	*N*-acetyl glucosamine-6-phospate	46.7	Arg 231	_	Arg 230
	*N*-acetyl glucosamine-1-phospate	36.7	Asp 70, Gln 58	_	Tyr 53
	*N*-acetyl glucosamine	39.6	Trp57, Asp70	Tyr61	
	Glucose	32.5	Trp57, Asp70, Tyr61, Tyr53	_	_
	Glucosamine	30.7	Tyr61, Asp70	_	_
**CviR (3QP4)**^∗^	DHL	58.8	Tyr80, Asp97, Trp84, Ser155, Met135	Val75, Leu85, Met89	_
	*N*-acetyl glucosamine-6-phospate	50.1	Ser155, Met135	Tyr88	_
	*N*-acetyl glucosamine-1-phospate	46.4	Thr140, Ser155, Trp84	Trp111, Tyr88	_
	*N*-acetyl glucosamine	38.9	Tyr80, Asp97	_	Tyr88
	Glucose	32.9	Trp84, Asp97	_	_
	Glucosamine	33.7	Trp84, Asp97	Trp111	_
**CviR (3QP5)**^∗^	Antagonist	54.8	Ser155, Asp97, Met135	Tyr88, Leu85, Met89	_
	*N*-acetyl glucosamine-6-phospate	48.4	Trp84, Tyr88, Asp97, Met135	Tyr80, Tyr88, Trp111	Tyr88
	*N*-acetyl glucosamine-1-phospate	47.7	Trp84, Tyr88,	Trp111	_
	*N*-acetyl glucosamine	39.9	Ser155, Asp97, Met135, Trp84	_	Tyr88, Ser155
	Glucose	33.5	Trp84, Asp97, Tyr88	Trp111	Ser155
	Glucosamine	33.5	Ser155, Asp 97, Tyr88	Trp111	Asp97

*^∗^Docking studies performed with two crystal structures, one bound to an agonist (3QP4) and another to an antagonist (3QP5). For others only agonist bound protein crystals are available.*

NAG-1-phosphate is predicted to form two out of the six hydrogen bonds (formed by OdDHL with the LasR AHL binding site in addition to three extra hydrogen bonds in the binding pocket. NAG-1-phosphate also forms one of four hydrogen bonds formed by *N*-octanoyl-L-homoserine lactone (OHL) in the TraR AHL binding site in addition to one extra bond and two of five or no shared hydrogen bonds formed by *N*-decanoyl-L-homoserine lactone (DHL) or an antagonist, respectively, with the CviR AHL binding site. The fitness score for NAG-6-phosphate is the highest among all analyzed ligands. NAG-6-phosphate is predicted to form two out of six hydrogen bonds formed by OdDHL with the LasR AHL binding site in addition to two extra hydrogen bonds. On the other hand, NAG-6-phosphate forms only one hydrogen bond (Arg 231) in the TraR binding site and has no shared hydrogen bonds formed by OHL in the binding pocket. NAG-6-phosphate also forms two of five or three of five hydrogen bonds formed by DHL or an antagonist, respectively, with the CviR AHL binding site. The results of this modeling investigation are congruent with our experimental data suggesting that low mM concentrations of NAG or phosphorylated NAG can interfere with transcription activated by LuxR type proteins.

### *N*-Acetylglucosamine Upregulates Extracellular Chitinase Activity of *C. violaceum* CV026

**FIGURE 6:**
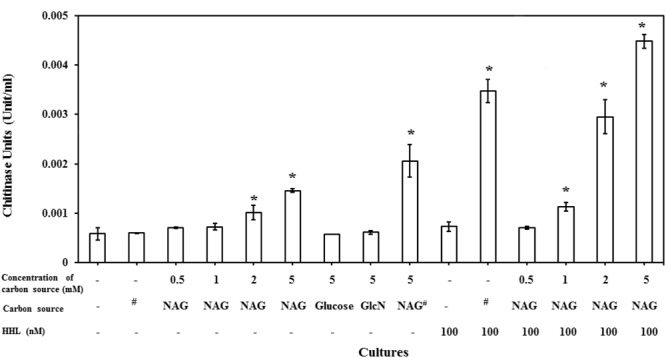
**NAG regulates extracellular chitinase activity of bacteria.** Concentration dependent effect of NAG on extracellular chitinase activity of *C. violaceum* (CV026) in the presence and absence of HHL 100 (nM). All cultures were in triplicates. Error bars represent standard deviation. Results represent the chitinase units/per cell. Asterisks indicate the significant differences in comparison to control samples (*P* < 0.01). “#” means chitin (1%).

*C. violaceum* CV026, which is known as an AHL synthase (*cviI*) deficient strain is reported to only produce chitinase activity on addition of AHLs (Chernin et al., 1998). It has also been reported that NAG can induce the expression of chitinase enzymes in different bacterial species. However, our results indicated that NAG inhibits QS-regulated violacein production of *C. violaceum* CV026. There is no report on the influence of NAG on extracellular chitinase activity in CV026. To test this, *C. violaceum* CV026 was cultured with various concentrations of NAG (0.5–5 mM) in the presence and absence of HHL (100 nM). **Figure 6** shows significant (*P* < 0.01) upregulation of the chitinase activity by 2–5 mM NAG even in the absence of exogenous HHL. In order to test whether this response was specific to NAG, chitin (1%), glucose (5 mM) or GlcN (5 mM) were examined revealing no induction of chitinase activity in the absence of HHL. In the presence of HHL (100 nM) chitinase activity increased in response to chitin (1%) addition. The strongest chitinase activity was observed in the presence of both NAG (5 mM) and HHL (100 nM). Similar results were observed with the *C. violaceum* wild type strain treated with increasing concentrations (0.5–5 mM) of NAG (**Supplementary Figure S1**). In summary, HHL upregulates violacein production and chitinase activity, whilst NAG upregulates chitinase activity but downregulates violacein production.

## Discussion

AHL dependent LuxR type proteins are one of many transcriptional regulators that are integrated to control expression of phenotypes in bacteria (Miller and Bassler, 2001). Other mechanisms that regulate transcription of genes influenced by LuxR type regulatory proteins include CRP, GroESL and Hnr (Shrout et al., 2006; Jahid et al., 2013). Results presented here suggest that NAG inhibits transcription of genes upregulated by AHL binding to LuxR, LasR and CviR based QS systems. NAG is the monomer of the second most abundant biopolymer on Earth and is also a major component of heterogeneous polysaccharides including murein and hyaluronic acid. It is commonly encountered in the environment in millimolar concentrations (Jagmann et al., 2012).

**FIGURE 7:**
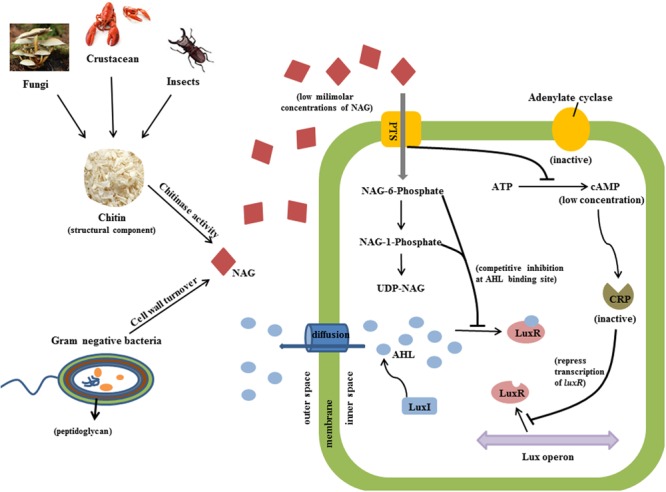
**Predicted inhibitory mechanism of NAG on QS activity is illustrated.** Chitinases cleave chitin from fungi, insects and crustacean and releases NAG molecules (red diamond). Peptidoglycan structure of bacteria is also an important source of NAG. Bacterial cells uptake NAG through membrane bounded phosphotransferases. High concentrations of NAG inhibit adenylate cyclase activity and thereby reduce the concentration of cAMP molecules which binds to CRP in order to induce transcription of *luxR*. Secondly, phosphorylated NAG molecules competitively bind to AHL binding site of LuxR protein at low milimolar concentrations and inhibit QS activity.

Several types of QS inhibitor compounds are produced by eukaryotes and prokaryotes and synthetic derivatives have shown QS inhibition activity (Wu et al., 2004). These compounds target QS systems by different mechanisms including inhibition of AHL production, enzymatic degradation of AHLs or interference with AHL binding or the stability of AHL receptors (Givskov et al., 1996; Zhang et al., 2002; Gao et al., 2003; Kalia, 2013; Chang et al., 2014). Evidence presented here suggests that NAG inhibits transcription of QS controlled genes via catabolite repression (CRP activity) and interference with some aspect of the QS mechanism (LuxR activity; **Figure 7**). Cellular uptake of NAG involves a phosphotransferase system that phosphorylates NAG as it enters the cell (Plumbridge, 1990). Therefore we speculate that phosphorylated NAG derivatives including NAG-1-phosphate and NAG-6-phosphate are likely responsible for the observed affects.

Phosphorylation of NAG could inhibit adenylate cyclase activity, which converts ATP into cAMP thereby influencing CRP transcriptional regulation activity (Kolb et al., 1993). The role of CRP in regulating carbon metabolism through carbon catabolite repression of *E. coli* and *B. subtilis* has been well characterized (Gorke and Stulke, 2008) but as mentioned above CRP also influences transcription of AHL dependent genes by binding to the same promoters of LuxR type proteins (Dunlap and Greenberg, 1988; Albus et al., 1997; Liang et al., 2007). Certainly, nutritional conditions have been shown to affect expression of QS regulated phenotypes (Shrout et al., 2006; Jahid et al., 2013). Assay inhibition by glucose and glucosamine presented here shows that the influence of catabolite repression is strain specific.

Molecular modeling is a commonly used and powerful technique to predict the binding affinity of a ligand to the binding site of a target protein where binding pockets have been defined by crystallography (Meng et al., 2011; Ferreira et al., 2015). It has been shown that molecular modeling is also an effective tool in the search for novel compounds with QS inhibitory activities (Yang et al., 2009; Ahumedo et al., 2010; Tan et al., 2013). From our docking studies, we found that NAG-1-phosphate and NAG-6-phosphate showed important hydrogen bonding interactions with LasR, TraR, and CviR proteins similar to their native AHL ligands. Receptor-ligand binding modeling suggests that NAG-1-phosphate and NAG-6-phosphate could inhibit transcription of AHL regulated genes by occupying the AHL binding site of LuxR type regulatory proteins. There are several non-AHL molecules that are capable of binding to AHL receptor proteins and interfering with native signal binding or increasing the turnover of receptor protein (LaSarre and Federle, 2013). For example, halogenated furanones are well known to compete with AHLs for binding to LuxR homologs resulting in a decrease in the concentration of the regulatory protein (Rasmussen et al., 2000; Manefield et al., 2002).

NAG can be utilized as a carbon/nitrogen source as well as regulate bacterial host colonization, production of virulence factors and biofilm formation (Chang et al., 2004; Korgaonkar and Whiteley, 2011; Jagmann et al., 2012; Kawada-Matsuo et al., 2012). In a previous study, transcriptome analysis to examine the gene expression levels of *P. aeruginosa* growing on NAG has shown that 5 mM NAG upregulates the abundance of quorum sensing repressor protein (QscR) sixfold (Korgaonkar and Whiteley, 2011). QscR is an orphan LuxR-type protein with no associated AHL synthase gene but responds to AHL molecules synthesized by LasI and RhlI (Chugani et al., 2001). Studies have shown that QscR negatively regulates the activities of both LasR and RhlR regulators through formation of heterodimers with receptor proteins and/or binding to their AHL molecules (Ledgham et al., 2003; Fuqua, 2006). Likewise, many other orphan LuxR-type regulatory genes are thought to exist within proteobacterial genomes and expression of these genes could also be regulated by NAG.

*P. aeruginosa* has a hierarchical QS-cascade between three separate QS circuits known as the Las, Rhl and PQS systems (Latifi et al., 1995; Pearson et al., 1995; Cao et al., 2001). Las and Rhl signaling systems regulate expression of OdDHL and BHL, respectively, while the PQS system regulates expression of the *Pseudomonas* quinolone signal molecule. It has been reported that low millimolar concentrations of NAG induce quinolone signal production in *P. aeruginosa* (Korgaonkar and Whiteley, 2011). Results presented in this study revealed that NAG inhibits the Las-dependent QS system, which is considered the master regulator of Rhl and PQS signaling systems (Pesci et al., 1997; McKnight et al., 2000). It is known that the QS regulatory cascade of *P. aeruginosa* is dependent on environmental and nutritional conditions (Dekimpe and Deziel, 2009; Cabeen, 2014). There are several examples of Las-independent regulation of Rhl and PQS mediated gene expression (D’Argenio et al., 2007; Bjarnsholt et al., 2010). For instance, starvation and phosphate-limitation induce transcriptional activation of Rhl and PQS in Las-deficient strains (Van Delden et al., 1998; Jensen et al., 2006).

NAG is widely used in dietary supplements, cosmetics and therapeutics development due to its unique features (Chen et al., 2010). It has been reported that NAG is a highly safe compound and intravenous injection of NAG (20 g) to the human body has no toxic effect and does not alter the blood glucose concentrations (Levin et al., 1961). Therefore, NAG is safely used as a nutritional supplement to treat diseases such as inflammatory bowel disease and osteoarthritis (Talent and Gracy, 1996; Kanazawa and Fukudo, 2006). On the other hand, there are many human pathogens such as *P. aeruginosa, S. marcescens* and *V. cholera*, which utilize QS to regulate their virulence activity (Zhu et al., 2002; Wei and Lai, 2006; Jimenez et al., 2012). The novel function of NAG on AHL-based QS activity can be used to develop therapeutic strategies to treat diseases associated with QS-mediated bacterial pathogenicity.

Additional tests investigated the relationship between NAG and HHL on extracellular chitinase activity of *C. violaceum* CV026. CV026 is reported to only produce chitinase and the purple pigment violacein on addition of AHLs (McClean et al., 1997; Chernin et al., 1998). Our results showed that NAG downregulates QS-regulated violacein production of *C. violaceum* CV026 while it upregulates QS-regulated extracellular chitinase activity. These results suggest that NAG influences extracellular chitinase activity through an unknown regulatory mechanism. This kind of inverse regulation has been recently reported on RhlR activity in *P. aeruginosa* which inversely modulates pyocyanin and rhamnolipid synthesis in response to non-native AHLs (Welsh et al., 2015).

The ability of bacteria to distinguish intra-cellular and inter-cellular NAG allows them to detect any possible threats from neighboring cells or pathogenic microorganisms (Konopka, 2012). Accordingly, bacteria could utilize NAG to switch on/off QS activity and modulate the production of QS-regulated phenotypes. This kind of a regulatory mechanism could also allow bacteria to avoid the cost of QS activity.

## Conclusion

This study demonstrates the effect of NAG on three different types of LuxR based QS systems. Evidence is presented indicating that NAG inhibits the QS activities of all analyzed strains. NAG is shown to repress transcription of genes upregulated by AHLs. Our results point out the possible involvement of both catabolite repression and competitive inhibition of AHL binding sites of LuxR type receptor proteins by phosphorylated NAG molecules. Inverse effects of NAG on regulation of violacein and extracellular chitinase synthesis in *C. violaceum* indicate that NAG could inhibit one QS regulated phenotype while simultaneously inducing another. Therefore, therapeutic applications of NAG require extra caution when targeting a certain type of a LuxR type receptor in order to avoid overexpression of undesirable phenotypes. Further research on identification of the role of NAG on QS regulation will improve our understanding of how bacteria survive in mixed communities within a host or in the environment and may help us to design novel therapeutic strategies to interfere with AHL dependent bacterial pathogenicity.

## Author Contributions

ÖK, SK, NK, and MM planned the experiments. ÖK, ZU, SN, ML, SK, and SB performed the experiments. ÖK, ZU, SB, and MM contributed to interpretation of data. ÖK, ZU, and MM contributed in writing the manuscript.

## Conflict of Interest Statement

The authors declare that the research was conducted in the absence of any commercial or financial relationships that could be construed as a potential conflict of interest.

## Supplementary Material

>The Supplementary Material for this article can be found online at: http://journal.frontiersin.org/article/10.3389/fmicb.2016.01313

FIGURE S1 **Concentration dependent effect of NAG on extracellular chitinase activity of *C. violaceum* WT strain.** All cultures were in triplicates. Error bars represent standard deviation. Results represent the chitinase units/per cell. Asterisks indicate the significant differences in comparison to control samples (*P* < 0.01).
