# Supplementary material for: N-Acetylglucosamine Inhibits LuxR, LasR and CviR Based Quorum Sensing Regulated Gene Expression Levels
Source: Front Microbiol. 2016 Aug 23;7:1313. doi: 10.3389/fmicb.2016.01313 (PMC4993992; doi:10.3389/fmicb.2016.01313)
Supplement: Supplementary file 1 [file Table_1.DOCX]

**Supplementary Table S1**. Bacterial strains and primers used in this study.

| **Strains/Primers** | **Relevant Characteristics** | **References** |
| --- | --- | --- |
| **Strains**  *Chromobacterium*  *violaceum* CV026  *Escherichia coli*  MT102  *Pseudomonas aeruginosa*  MH602  **Primers**  vioA F  vioA R  vioC F  vioC R  vioD F  vioD R  gfp(ASV) F  gfp(ASV) R | *cviI::*mini-Tn5 derivative of ATCC  31532, Kan^R^, acyl-HSL^-^  ( 50 µg/ml kanamycin)  *E. coli* cells harbouring *p*JBA132  expressing Gfp(ASV) in response to AHL  Amp^R^  (100 µg/ml ampicillin)  *Pseudomonas* shuttle vector carrying  P*_las_*_B_-gfp(ASV) P*_lac_*-*lasR*;Amp^R^, Gm^R^  (20 µg/ml gentamicin)  5'-GCT GAA AGA GCA TGG CAA GG-3'  5'-GTC GTT ATC GGT CAC GCT CT-3'  5'-CTC AGC TGC TCC AAT CCC AT-3'  5'-TCT TCA TCA GTT GAC CCT CCC T-3'  5'-GAA GAC TTC AAG CTG GTC CAC-3'  5'-ATA GGT CTC CTC GCT GCA CT-3'  5'-TGG AAA CAT TCT TGG ACA CA-3'  5'-ACC ATG TGG TCT CTC TTT TC-3' | (McClean et al., 1997)  (Andersen et al., 2001)  (Hentzer et al., 2002)  This study |
