# Supplementary figures and images for: N-Acetylglucosamine Inhibits LuxR, LasR and CviR Based Quorum Sensing Regulated Gene Expression Levels
Source: Front Microbiol. 2016 Aug 23;7:1313. doi: 10.3389/fmicb.2016.01313 (PMC4993992; doi:10.3389/fmicb.2016.01313)

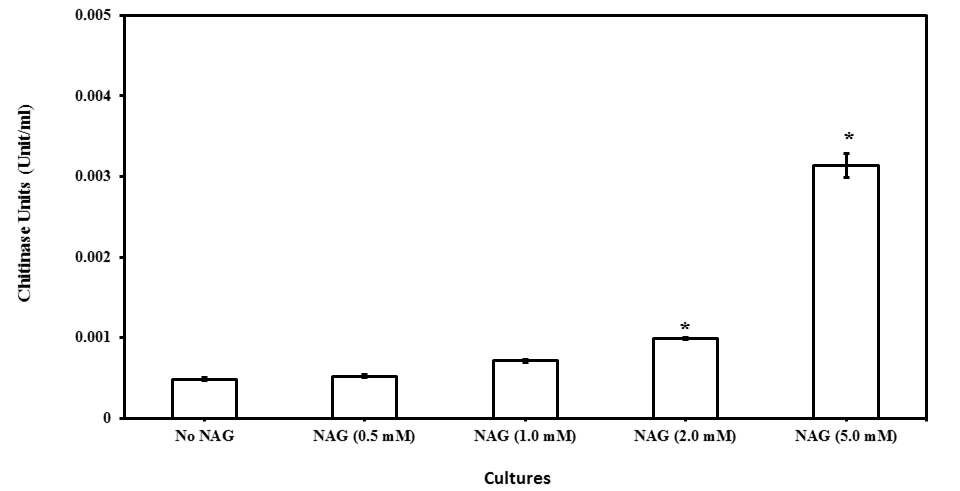

Supplement: FIGURE S1 — Concentration dependent effect of NAG on extracellular chitinase activity of C. violaceum WT strain. All cultures were in triplicates. Error bars represent standard deviation. Results represent the chitinase units/per cell. Asterisks indicate the significant differences in comparison to control samples (P < 0.01). [file Image_1.TIF]
